# Autophagy Is Required to Sustain Increased Intestinal Cell Proliferation during Phenotypic Plasticity Changes in Honey Bee (*Apis mellifera*)

**DOI:** 10.3390/ijms24031926

**Published:** 2023-01-18

**Authors:** Yueqin Guo, Ruoyang Hu, Naikang Li, Nannan Li, Jiangli Wu, Huimin Yu, Jing Tan, Zhouhua Li, Shufa Xu

**Affiliations:** 1 Key Laboratory of Pollinating Insect Biology, Ministry of Agriculture and Rural Affairs, Institute of Apicultural Research, Chinese Academy of Agricultural Sciences, Beijing 100193, China; 2 School of Life Sciences, Capital Normal University, Beijing 100048, China

**Keywords:** honey bee (*Apis mellifera*), autophagy, phenotypic plasticity, midgut, cell proliferation

## Abstract

Tissue phenotypic plasticity facilitates rapid adaptation of organisms to biotic and/or abiotic pressure. The reproductive capacity of honey bee workers (*Apis mellifera*) is plastic and responsive to pheromones produced by broods and the queen. Egg laying workers (ELWs), which could reactivate their ovaries and lay haploid eggs upon queen lost, have been commonly discussed from many aspects. However, it remains unclear whether midgut homeostasis in ELWs is affected during plastic changes. Here, we found that the expression of nutrition- and autophagy-related genes was up-regulated in the midguts of ELWs, compared with that in nurse workers (NWs) by RNA-sequencing. Furthermore, the area and number of autophagosomes were increased, along with significantly increased cell death in the midguts of ELWs. Moreover, cell cycle progression in the midguts of ELWs was increased compared with that in NWs. Consistent with the up-regulation of nutrition-related genes, the body and midgut sizes, and the number of intestinal proliferation cells of larvae reared with royal jelly (RJ) obviously increased more than those reared without RJ *in vitro*. Finally, cell proliferation was dramatically suppressed in the midguts of ELWs when autophagy was inhibited. Altogether, our data suggested that autophagy was induced and required to sustain cell proliferation in ELWs’ midguts, thereby revealing the critical role of autophagy played in the intestines during phenotypic plasticity changes.

## 1. Introduction

Phenotypic plasticity, the ability of organisms to change their morphology and behavior without altering their genotype, facilitates rapid adaptation in response to biotic and/or abiotic pressure [1,2,3]. During this process coordinated control of global transcription and epigenetic regulation of the genome are required [1,4]. Eusocial insects exhibit dramatic phenotypic plasticity with morphologically distinct reproduction capabilities between queen and worker castes [5,6]. In a honeybee (*Apis mellifera*) hive, young larvae of the honeybees are totipotent; they can become either queens (reproductive) or workers (largely sterile workers). The caste differentiation is determined by the environment and diet of young larvae, besides genotype [4,7,8]. The queens are fed with a diet exclusively composed of royal jelly (RJ), which plays essential roles in driving queen development, caste determination, and reproduction capacity [9,10,11]. The reproductive capacity of honeybee workers is plastic and responsive to pheromones [12,13], which not only inhibit ovary activation and reproductive behavior in worker bees, but also induce young workers to feed and groom the queen and to perform colony-related tasks [14,15]. Queen pheromone triggers programmed cell death at the mid-oogenesis checkpoint causing the abortion of worker oocytes and reproductive inhibition of the worker caste [16]. When the queen is absent for several days, the portion of workers with active ovaries is obviously increased, with some workers being capable of laying eggs [17,18]. Both larval and adult nutrition have effects on worker honeybee ovary development and body mass [19]. If adult workers are forced to consume RJ in an artificial diet, they will develop a more queen-like phenotype, including ovary activation and increased lifespan [20,21]. During the plastic activation process the worker ovary is completely remodeled to develop into different types of cells, producing oocytes, switching on vitellogenesis [22], and finally producing eggs [1]. Notch signaling in the germarium of worker bee ovaries is important for reproduction activation in the worker [18]. Despite significant progress in the study of plastic changes, the underlying mechanism remains largely unknown.

In insects autophagy participates in many physiological processes including cell proliferation, organism development, and response to starvation and pathogen infection [23,24]. In eukaryotic cells autophagy is a highly conserved and regulated process, in which cytosolic materials, including organelles, are sequestered within double-membrane vesicles termed autophagosomes [24]. Autophagy remodels cytoplasm, recovers essential nutrients, and disposes of unwanted cytoplasmic components [24,25]. More than 40 autophagy-related (ATG) proteins participate in the nucleation, elongation, and maturation of the autophagosome membrane [24,26], and most of these genes are highly conserved from yeast to mammals [27,28,29]. In holometabolous insects, many tissues and organs such as the midgut and fat body undergo a remodeling process during metamorphosis [30,31,32]. In sand flies, morphological changes in the midgut epithelium are paired with the up-regulation of *atg1*, *atg6*, and *atg8* during the larva-adult transition [33]. In silkworm *Bombyx mori*, autophagy plays important functions in the process of larval stem cells differentiating into pupal midgut epithelium [34]. During the pupal period of *Galleria*, autophagosomes form in parallel with the up-regulation of *atg6* and *atg8* in the fat body [35]. In the hindgut epithelium of honeybee prepupae, many autophagosomes are evidenced by the immunolocalization of LC3-proteins [36]. Autophagy has been also reported to affect stem cell maintenance and differentiation in the remodeling processes [37]. These findings suggest that autophagy plays important functions during metamorphosis and development in insects. However, whether autophagy is activated and required in the midgut of egg laying workers (ELWs) during plastic changes is not clear.

The autophagy activity is regulated by two major kinases, including mechanistic target of rapamycin kinase (mTOR) and the class III PtdIns3K (phosphatidylinositol 3-kinase, PI3K) complex. Autophagy can be strongly inhibited by mTOR, which activates protein biosynthesis and cell growth. Meanwhile, autophagy can be stimulated by the class III PtdIns3K complex, which facilitates the synthesis of PtdIns3P at the phagophore assembly site membranes [38]. The 3-methyladenine (3-MA), which is a PI3K and PtdIns3K inhibitor, suppresses autophagic signaling by inhibiting the VPS34/PI3K complex [39]. Bafilomycin A1 (BAF) inhibit autophagosome-lysosome fusion by increasing the pH level of the lysosome, and BAF is a late-stage inhibitor [40]. Here, we have examined the intestinal cell proliferation after ELWs were treated with autophagy inhibitors, 3-MA and BAF.

The honeybee midgut is involved in food digestion, nutrient absorption, and is responsible for immunity and detoxification [41,42]. The midgut is lined with the peritrophic matrix, which is a protective barrier. Stem cell self-renewal is known to maintain the intestinal epithelium in other insects such as *Drosophila* and silkworm [43,44]. In *Drosophila melanogaster*, intestinal stem cells (ISCs) are located adjacent to the basement membrane of the midgut epithelium [45,46,47]. In silkworm *Bombyx mori*, stem cell proliferation and differentiation exist in the larval, pupal and adult midgut epithelium [34,48]. The intestinal cell proliferation is abundant and showed an age-related decline in workers, queens, and drones, and queen intestinal stem cells have a relatively high replicative potential [49]. In the queen-less colony, previous studies mostly focused on the ovary reactivation of queen-less workers. Because of the essential biological function of midguts, the intestinal homeostasis of ELWs may alternate during phenotypic plasticity change. Autophagy have been confirmed to participate in multiple biological processes, but whether autophagy influenced the intestinal homeostasis of ELWs was unclear. Whether and how intestines are remodeled during ELWs phenotypic plasticity changes also remain unknown.

In this study, we focused on the transcriptional, physiological, and structural differences in midguts between nurse workers (NWs) and ELWs during plastic changes. The transcriptome profiling results showed that the expressions of important genes and signaling pathways were increased in ELWs, especially the nutrition and autophagy related genes. Consistently, the number and size of autophagosomes and cell proliferation were significantly increased in the midgut of ELWs. Importantly, increased cell proliferation was dramatically suppressed by autophagy inhibition. Thus, our data uncover that autophagy plays a critical role in intestines of ELWs during phenotypic plasticity changes.

## 2. Results

### 2.1. Key Genes and Signaling Pathways Are Affected in the Midguts of ELWs during Phenotypic Plasticity

**Figure 1:**
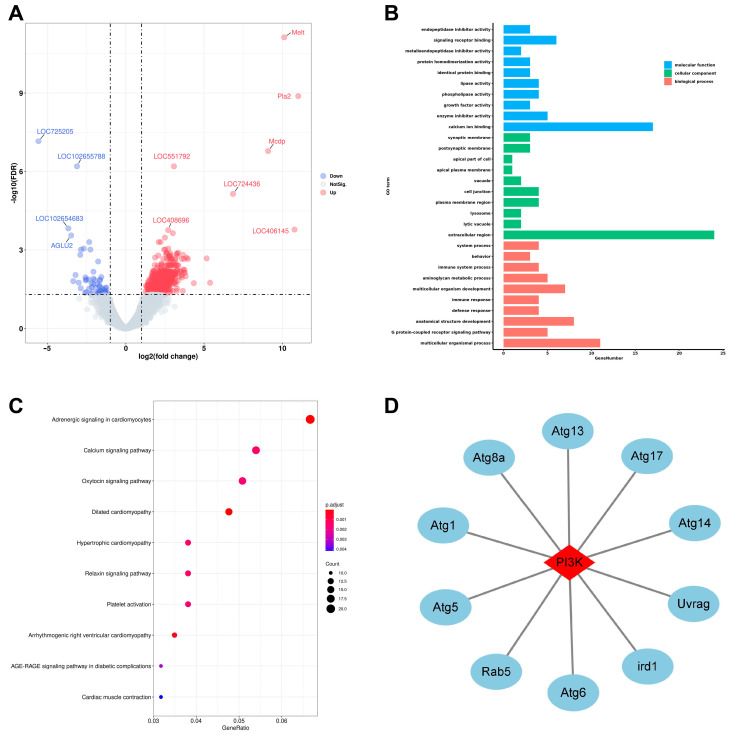
Key genes and signaling pathways are affected in the midguts of egg laying workers (ELWs). (**A**) Volcano plots of differentially expressed genes between the midguts of nurse workers (NWs) and ELWs (up-regulated genes were signed by red dots and down-regulated genes were signed by blue dots). Each dot represents one gene. The gray dots represent genes that were not differentially expressed. (**B**) Enrichment of DEGs among the GO terms in the biological process, cellular component, and molecular function categories. (**C**) Bubble chart of KEGG pathways enriched by up-regulated DEGs in ELWs’ midguts. (**D**) Protein interaction network of DEGs encoding proteins related to PI3K in the midguts of NWs_vs._ELWs. PI3K interacts with autophagy-related genes.

In order to examine whether honey bee midgut homeostasis was affected during phenotypic plasticity, gene expression profiling in the midguts of NWs and ELWs was characterized by RNA-seq. A total of 723 differentially expressed genes (DEGs) were identified between the midguts of NWs and ELWs: in ELWs, 668 (92.4%) genes were up-regulated, and 55 (7.6%) genes were down-regulated (Figure 1A). The GO terms analysis showed that these DEGs were involved in diverse biological processes, including ion binding, enzyme activity, membrane and extracellular region location, multicellular organismal process, and development (Figure 1B).

The DEGs were also mapped to canonical KEGG pathways to identify possible active biological pathways. Many DEGs were up-regulated and related with autophagy (Figure 1C, Table S2). Most of these DEGs were involved in signaling pathways such as calcium signaling pathway [50], AGE-RAGE signaling pathway in diabetic complications [51], aldosterone synthesis and secretion [52], platelet activation [53], melanogenesis [54], pancreatic secretion [55], GnRH signaling pathway [56], Sphingolipid signaling pathway [57], cAMP signaling pathway [58], thyroid hormone signaling pathway [59], MAPK signaling pathway [60], insulin secretion [61], and so on. Of note, some of the DEGs were concentrated on nutritional absorption, including starch and sucrose metabolism [62], protein digestion and absorption [63], and carbohydrate digestion and absorption [64], suggesting that these DEGs participate in the control of reproductive potential in response to nutritional stress [11]. We hypothesized that the variation in the expression of these genes might stimulate the nutritional absorption in midguts, which may provide more nutrition and energy for ELWs to undergo plastic changes and lay eggs.

Except GO and KEGG analysis, the interaction networks of corresponding proteins were predicted using STRING among these DEGs. Results showed that some key genes related to autophagy were up-regulated in ELWs. Among them, PI3K (LOC408577), expressed 3.0-fold up-regulated in midguts of ELWs than in NWs (Table S1). PI3K interacted with autophagy-related genes (Figure 1D), and the PI3K/Akt/mTOR signaling is important for autophagy initiation [65]. These data suggested that the phenotypic plastic change might be related with autophagy in midguts of ELWs.

### 2.2. Key DEGs Are Validated in the Midguts of ELWs

**Figure 2:**
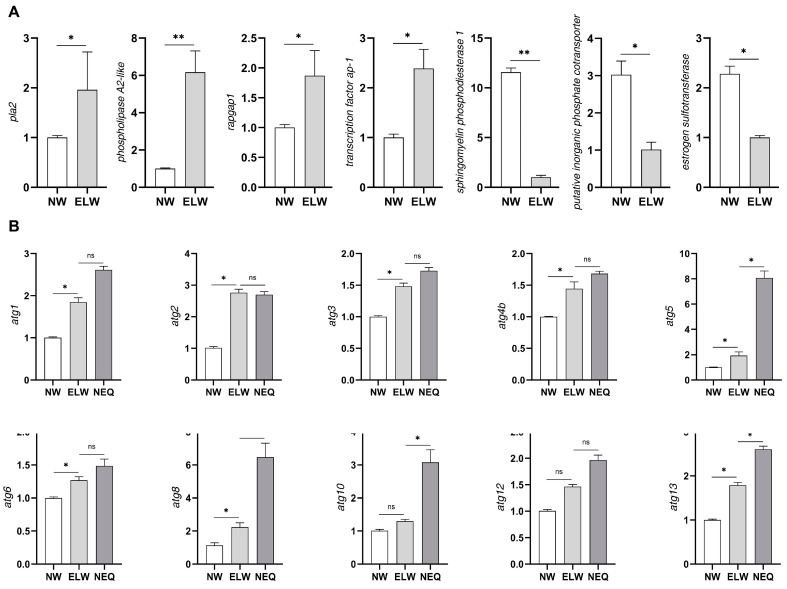
Validation of key DEGs in the midguts of ELWs. (**A**) Expression profile of transcripts that were differentially expressed between the midguts of NWs and ELWs determined by qRT-PCR. Significant differences between the midguts of NWs and ELWs were determined by Student’s *t*-test. Asterisks indicate significant differences. * *p* < 0.05; ** *p* < 0.01. (**B**) The expression of autophagic genes was significantly increased in the midguts of ELWs. qRT-PCR quantification of autophagy genes (*atg*), including *atg1*, *2*, *3*, *4b*, *5*, *6*, *8*, *10*, *12*, and *13*, from the whole midguts of NWs, ELWs, and newly emerged queens (NEQs). Ribosomal gene *rp49* was used as normalization control. Means ± SEM are shown. *n* = 3. Significant differences between the midguts of NWs, ELWs, and NEQs were determined by one way analysis of variance (ANOVA)/Dunn’s Method. Asterisks indicate significant differences. * *p* < 0.05; ns *=* not significant.

The expression of four up-regulated DEGs were further validated using qRT-PCR. The expression of phospholipase A2 (*pla2*) and phospholipase A2-like (LOC724436) was obviously up-regulated (Figure 2A). Both genes are related to lipid transport and localization, and expressed in the reproductive tract in *Drosophila* [66]. The up-regulated expression of Rap1 (Ras-associated protein 1) GTPase-activating protein 1 (*rapgap1*), and transcription factor *ap-1* (LOC726289) was also confirmed by qRT-PCR (Figure 2A and Table S1). Rapgap1 is a negative regulator of Rap1-mediated signaling [67]. Rap1 (Ras-associated protein 1) is an essential regulator of morphogenesis [68] and participates in autophagy [69]. AP-1/Fos and EGFR/MAPK signaling stimulate autophagy [37]. AP-1 interacts with ATG9A, which is the essential regulator of autophagy [70]. The up-regulation of *rapgap1* and *ap-1* may influence autophagy in ELWs’ intestines.

The expression of three down-regulated DEGs was also confirmed by qRT-PCR in midguts of ELWs, including estrogen sulfotransferase (LOC411376), sphingomyelin phosphodiesterase 1 (LOC726315), and putative inorganic phosphate cotransporter (LOC413263). Estrogens are sulfated and inactivated by estrogen sulfotransferase, which can regulate estrogens homeostasis [71]. The expression of estrogen sulfotransferase was decreased in the ELWs’ midguts (Figure 2A), which may result in the stimulation of estrogens, and then influence ELWs in egg laying. In bumblebee, sphingomyelin phosphodiesterase might play an important role in female oviposition [72]. In *Drosophila* ovary, the high transcript levels of inorganic phosphate cotransporter were observed in the nurse cells and transferred to the oocyte [73]. It suggested that putative inorganic phosphate cotransporter might have essential functions in ovary homeostasis. The expression of *smpd1* and inorganic phosphate cotransporter was down-regulated in the ELWs’ midguts (Figure 2A), which may have some direct or indirect influence on egg laying of ELWs.

In addition to those seven DEGs we found that some genes that encoding NF-kappa-B inhibitor cactus (LOC411012, 2.7-fold), trehalase isoform X1 (LOC410795, 2.4-fold), ribosomal protein S6 kinase alpha-5 (LOC411630, 2.3-fold), stress-activated protein kinase JNK (LOC409286, 2.4-fold), TGF-beta receptor type-1 isoform X9 (LOC550930, 2.5-fold), cAMP-dependent protein kinase catalytic subunit (LOC409791, 2.2-fold), adenylate cyclase type 2 (LOC552216, 1.6-fold), and G protein alpha q subunit isoform X5 (LOC550818, 3.1-fold) were up-regulated in midguts of ELWs compared with those in NWs (Table S1). Previous reports showed that these genes directly or indirectly regulated autophagy pathway in previous research [58,74,75]. These data implied that the phenotypic plasticity was related with autophagy.

### 2.3. Autophagy Genes Are Up-Regulated in the Midguts of ELWs during Plastic Changes

To further confirm our speculation, we performed qRT-PCR to examine whether the expression of autophagy genes was altered during plastic changes. We measured the transcript levels of different autophagic genes, including *atg1*, *2*, *3*, *4b*, *5*, *6*, *8*, *10*, *12*, and *13*, in the midguts of NWs, ELWs, and newly emerged queens (NEQs). Significant increase in the expression of all examined autophagy genes was observed in the midguts of ELWs during plastic changes, compared with that in the midguts of NWs (Figure 2B). Interestingly, the expression of these autophagic genes was similar to that of NEQs, albeit the levels of some autophagic genes in the midguts of ELWs were lower than those in the NEQs, indicating that these ELWs may undergo a plastic change process (Figure 2B). These results indicated that the autophagy may be induced in the midguts of ELWs during the process of phenotypic plasticity changes.

### 2.4. Autophagy Is Activated in the Midgut of ELWs

**Figure 3:**
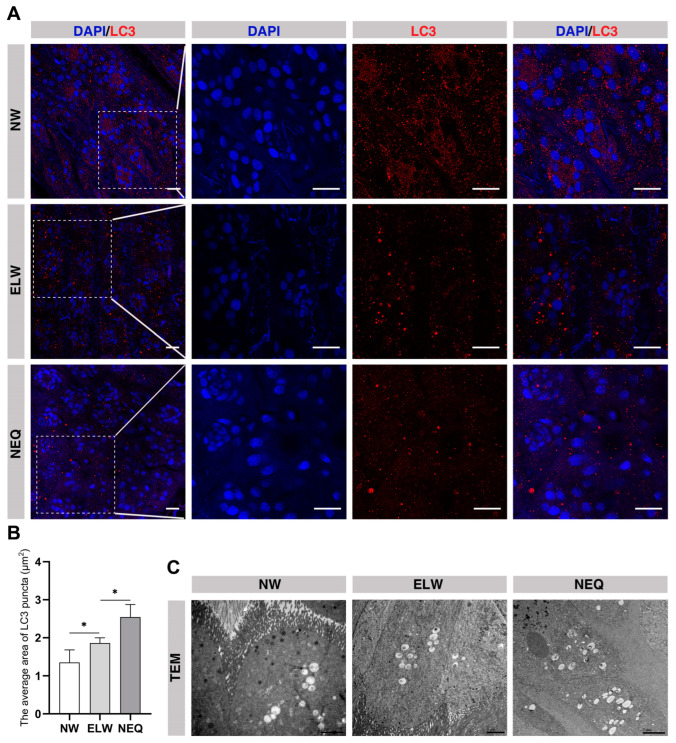
Autophagosomes are significantly increased in the midguts of ELWs. (**A**) LC3 antibody staining in the midguts of NWs, ELWs, and NEQs. The confocal images of 40 × lens/1.0 zoom of NWs, ELWs, and NEQs were demonstrated on the left column, and the confocal images of 40 × lens/2.0 zoom were demonstrated on the right three columns. In all the panels except graphs, blue indicates DAPI staining, and red indicates LC3 staining. Scale bars, 25 µm. (**B**) Quantification of the LC3 puncta areas in midguts of workers and queens in (**A**) (NWs, 1.34 ± 0.04 μm^2^, *n* = 12 intestines; ELWs, 1.86 ± 0.14 μm^2^, *n* = 13 intestines; newly emerged queen, 2.55 ± 0.33 μm^2^, *n* = 12 intestines). Means ± SEM are shown. Significant differences between the midguts of NWs, ELWs, and NEQ were determined by one way ANOVA/Dunn’s Method. Asterisks indicate significant differences between the midguts of NWs, ELWs, and QLWs. * *p* < 0.05. (**C**) TEM images from the midguts of NWs, ELWs, and NEQs. Scale bar = 2 µm.

To further confirm the notion that the autophagic genes were up-regulated and autophagy was activated in the midguts of ELWs, we examined the ATG8/LC3 (the widely used autophagic indicator) levels using the LC3A/B antibody in midguts of NWs, ELWs, and NEQs (Figure 3). This rabbit anti-LC3A/B antibody was used to identify autophagosomes in post-embryonic malpighian tubules in *Apis mellifera* [76]. In the midguts of NWs, only a few LC3^+^ puncta (autophagosomes/autolysosomes) were dispersedly localized in each crypt (Figure 3A). A significant increase in the size of autophagosomes was observed in the intestines of ELWs, compared with that in NWs (NWs, 1.34 ± 0.04 μm^2^; ELWs, 1.86 ± 0.14 μm^2^) (Figure 3A,B). Consistent with the qRT-PCR results, the levels of ATG8 and the size of autophagosomes in the intestines of ELWs were close to those in the midguts of the NEQs (2.55 ± 0.33 μm^2^) (Figure 3B). The results showed that the levels of LC3 and LC3 puncta in the midguts of ELWs were significantly enhanced compared to those in NWs. The enhanced expression of *atg* genes and increased accumulation of LC3-positive puncta supported increased induction of autophagy in the midguts of ELWs.

To further confirm this notion, transmission electron microscopy analysis was performed on honey bee midguts. The average number of autophagosomes in ELWs’ midguts was clearly increased, compared with that in NWs. The average number of autophagosomes was similar to that in the NEQs (Figure 3C). These results suggested that autophagy was likely involved in ELWs’ midguts during the process of phenotypic plasticity.

### 2.5. Autophagy-Induced Cell Death Is Increased in the Midguts of ELWs

**Figure 4:**
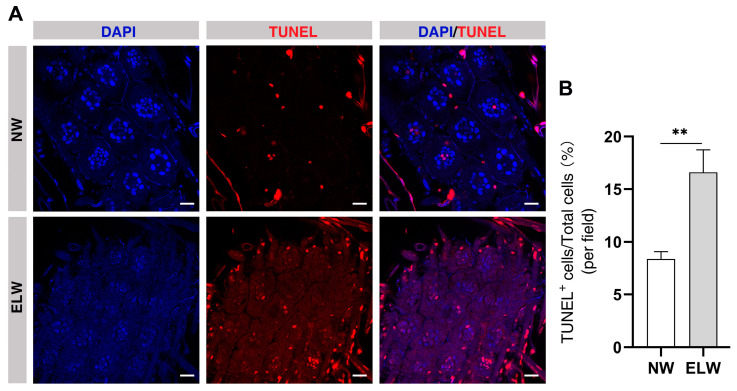
Cell death is significantly increased in the midguts of ELWs. (**A**) Detection of programmed cell death by TUNEL assay in the midguts of NWs and ELWs. In all the panels except graphs, blue indicates DAPI staining, and red indicates TUNEL-positive cells. Scale bars, 25 µm. (**B**) The percentage of TUNEL-positive cells in midguts of NWs and ELWs (NWs, 8.37 ± 0.70%, *n* = 12 intestines; ELWs, 16.61 ± 2.13%, *n* = 14 intestines). Means ± SEM are shown. Significant differences between the midguts of NWs and ELWs were determined by Student’s *t*-test. Asterisks indicate significant differences between the midguts of NWs and ELWs. ** *p* < 0.01.

Increased autophagy induction often results in increased cell death. In order to elucidate the mechanism of phenotypic plasticity by autophagic induction in midguts, we detected cell death using TUNEL-labeling experiments between NWs and ELWs (Figure 4). In the midguts of NWs, dying cells (TUNEL-positive) were observed in the base of crypt and outside of the crypt (Figure 4A). A significant increase in TUNEL-positive cells was observed in intestines of ELWs compared with that in NWs (NWs, 8.37 ± 0.70%; ELWs, 16.61 ± 2.13%). Most of the TUNEL-positive cells were localized in the base of crypt, with some dying cells localized outside of the crypt (Figure 4A,B). These data suggested that a higher cellular turnover rate in the midguts of ELWs and the high turnover rate may be a direct consequence of autophagy induction during plastic changes. However, we cannot exclude the possibility that increased apoptosis is independent of increased autophagy induction in these intestines, and also plays a role in intestinal homeostasis during plastic changes in ELWs.

### 2.6. Cell Proliferation Is Significantly Increased in the Midguts of ELWs

**Figure 5:**
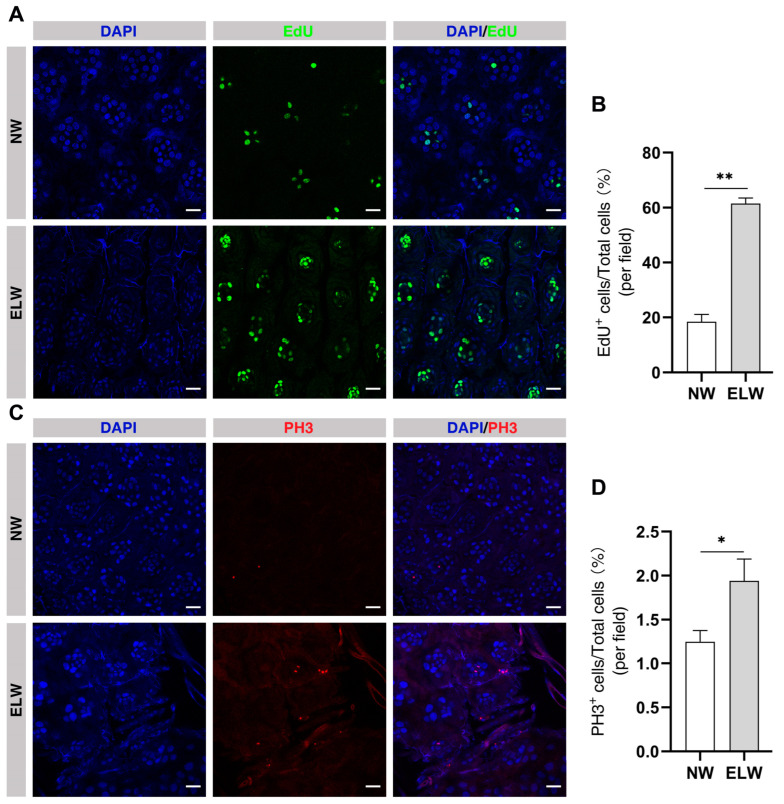
Cell proliferation is significantly increased in the midguts of ELWs. (**A**) Detection of cell proliferation by Click-it EdU assay in the midguts of NWs and ELWs. In all the panels except graphs, blue indicates DAPI staining, and green indicates EdU-positive cells. Scale bars, 25 µm. (**B**) The percentage of EdU-positive cells in the midguts of NWs and ELWs (NWs, 18.46 ± 0.87%, *n* = 12 intestines; ELWs, 61.49 ± 2.00%, *n* = 13 intestines). Means ± SEM are shown. (**C**) Detection of cell proliferation with PH3 antibody in the midguts of NWs and ELWs. In all the panels except graphs, blue indicates DAPI staining, and red indicates PH3-positive cells. Scale bars, 25 µm. (**D**) The percentage of PH3-positive cells in the midguts of NWs and ELWs (NWs, 1.25 ± 0.13%, *n* = 10 intestines; ELWs, 1.94 ± 0.25%, *n* = 12 intestines). Means ± SEM are shown. Significant differences between the midguts of NWs and ELWs were determined by Student’s *t*-test. Asterisks indicate significant differences between the midguts of NWs and ELWs. * *p* < 0.05, ** *p* < 0.01.

Autophagic degradation can provide cells with more nutrients for survival and proliferation. To examine whether increased autophagy in intestines of ELWs was related to cell proliferation, we examined cell proliferation in these intestines. The mitotic marker bromo-deoxyuridine (BrdU) was used to compare the proliferative activity of intestinal stem cells among queens, workers, and males of different ages in honey bees (*Apis mellifera* L.) [49]. Compared with BrdU, ethynyl deoxyuridine (EdU) was faster and easier to label dividing cells. We first detected the cell proliferation using Click-iT EdU cell proliferation Assay Kit in the intestines of NWs and ELWs. The percentage of EdU-positive cells in midguts of ELWs was significantly increased compared with that in the midgut of NWs (NWs, 18.46 ± 0.87%; ELWs, 61.49 ± 2.00%) (Figure 5A,B). In the midguts of NWs, EdU-positive cells were regularly distributed in the base of the crypt and the number of EdU-positive cells in each crypt was about four (Figure 5A). While the number of EdU-positive cells was significantly increased in every crypt of ELWs, indicating a significant increase in cells in S stage (Figure 5A,B). Endopolyploidy is commonly observed in midguts of many insects [77]. Endopolyploidy nuclei derive from endoreduplication, in which DNA of the chromosomes is normally replicated but without mitotic division; in the end the sister chromatids do not divide [78]. In the midguts of ELWs the colocalization of EdU and LC3 was detected (Figure S1). In replicating chromosomes, EdU was integrated into newly synthesized DNA. The signal of ATG8/LC3 was almost dispersed in cytoplasm of ELWs midgut. Both cell proliferation and autophagy occurred in ELWs midguts (Figure S1).

PH3 antibody (an endogenous cell cycle marker) was used to further label cells in mitosis. Consistent with the EdU results, the percentage of PH3-positive cells in the midguts of ELWs was significantly increased compared with that in NWs (NWs, 1.25 ± 0.13%; ELWs, 1.94 ± 0.25%) (Figure 5C,D). Collectively, these data supported the notion that cells underwent increased proliferation with the help of nutrients recycled by increased autophagy in the midguts of ELWs during plastic changes.

### 2.7. The Intestinal Cell Proliferation of Honey Bee Larvae Is Influenced by the Nutritional Level

**Figure 6:**
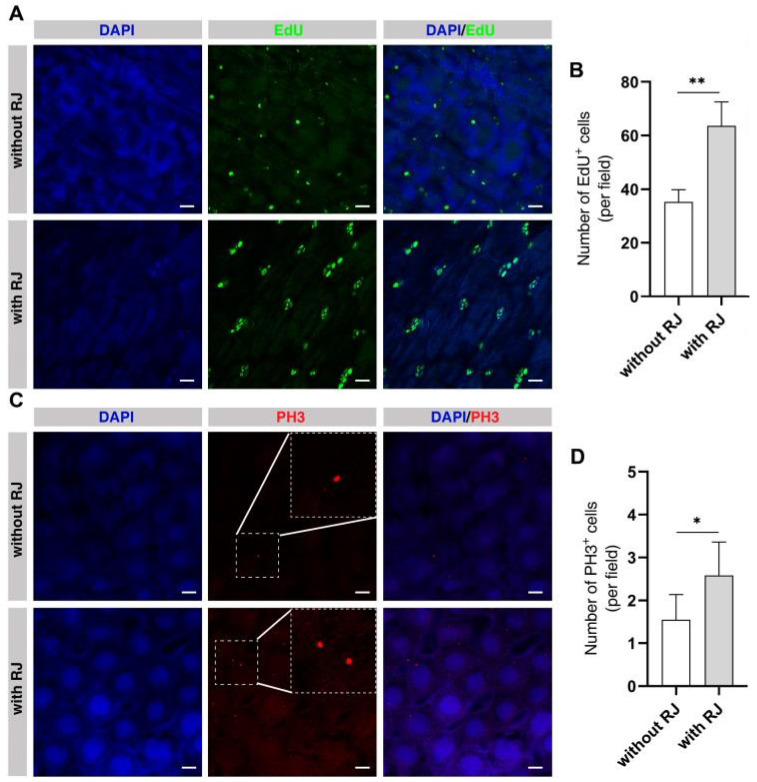
RJ affects intestinal homeostasis of larvae. (**A**) Detection of cell proliferation by Click-it EdU assay in the midguts of larvae reared with or without RJ. In all the panels except graphs, blue indicates DAPI staining and green indicates EdU-positive cells. Scale bars, 25 µm. (**B**) Quantification of EdU-positive cells in the midguts of larvae reared with or without RJ (without RJ, 35.29 ± 1.21, *n* = 12 intestines; with RJ, 67.08 ± 3.27, *n* = 10 intestines). (**C**) Detection of cell proliferation with PH3 antibody in the midguts of larvae reared with or without RJ. In all the panels except graphs, blue indicates DAPI staining and red indicates PH3-positive cells. Scale bars, 25 µm. (**D**) Quantification of PH3-positive cells in the midgut of larvae reared with or without RJ (without RJ, 1.55 ± 0.13, *n* = 12 intestines; with RJ, 2.58 ± 0.16, *n* = 10 intestines). Means ± SEM are shown. Significant differences between the midguts of larvae with or without RJ were determined by Student’s *t*-test. Asterisks indicate significant differences between the midguts of larvae with or without RJ. * *p* < 0.05, ** *p* < 0.01.

Based on the above-mentioned data, we hypothesized that in the absent queen colony the ELWs have the different nutritional status with those NWs, and autophagy is induced to recycle nutrients to sustain phenotypic plasticity. To further confirm this hypothesis, we examined the effect of the nutrients on intestinal cell proliferation by rearing honey bee larvae with RJ or without RJ in artificial food. We examined cell proliferation using EdU assay and PH3 antibody in the midguts of honey bee larvae reared with and without RJ. The body and midgut sizes of larvae reared with RJ were much larger, while those reared without RJ grew very poorly (Figure S2). The relative number of EdU-positive cells in midgut of larvae reared with RJ was significantly increased than that in the midgut of larvae without RJ (without RJ, 35.29 ± 1.21; with RJ, 67.08 ± 3.27) (Figure 6A,B). Some large positive EdU cells may be also endopolyploid in midgut of honey bee larvae. Consistently, the number of PH3-positive cells in the midgut of larvae reared with RJ was significantly increased from that in the midgut of larvae without RJ (without RJ, 1.55 ± 0.13; with RJ, 2.58 ± 0.16) (Figure 6C,D). This mimicked that of ELWs during phenotypic plasticity. These results indicated that nutritional status had important impact in cell proliferation in midgut.

### 2.8. Autophagy Is Required in the Midguts of ELWs during Phenotypic Plasticity Change

**Figure 7:**
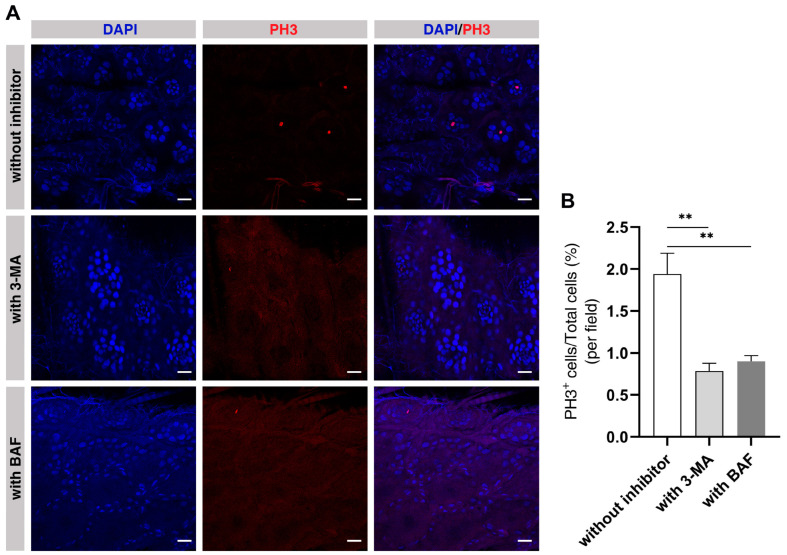
Inhibition of autophagy almost completely suppresses cell proliferation in the midguts of ELWs. (**A**) Detection of cell proliferation with PH3 antibody assay in the midguts of ELWs which were fed with or without autophagy inhibitors, such as 3-MA and BAF. In all the panels except graphs, blue indicates DAPI staining, and red indicates PH3 staining. Scale bars, 25 µm. (**B**) The percentage of PH3-positive cells in the midguts of ELWs fed with or without autophagy inhibitors (without autophagy inhibitor, 1.94 ± 0.24%, *n* = 10 intestines; with 3-MA, 0.79 ± 0.09%, *n* = 10 intestines; with BAF, 0.90 ± 0.07%, *n* = 11 intestines). Means ± SEM are shown. Significant differences between the midguts of ELWs fed without or with autophagy inhibitors were determined by one way ANOVA/Dunn’s Method. Asterisks indicate significant differences between the midguts of ELWs fed without or with autophagy inhibitors. ** *p* < 0.01.

We further examined whether the above-mentioned phenotypes during plastic changes were regulated by autophagy. Autophagy can be effectively blocked by the well-known autophagy inhibitors, 3-methyladenine (3-MA, a PI3K and PtdIns3K inhibitor) and Bafilomycin A1 (BAF, a vacuolar-type H^+^-translocating ATPase inhibitor) [39,40]. ELWs were treated with 3-MA or BAF to inhibit autophagy. Interestingly, the percentage of PH3-positive cells was dramatically decreased in midguts with autophagy inhibition using the autophagy inhibitors compared to that in ELWs without 3-MA or BAF administration (without autophagy inhibitor, 1.94 ± 0.24%; with 3-MA, 0.79 ± 0.09%; with BAF, 0.90 ± 0.07%) (Figure 7A,B). NWs were also treated with 3-MA to inhibit autophagy. There was no obvious difference in intestinal cell proliferation of NWs treated with or without 3-MA (Figure S3). The 3-MA or BAF treatment had no or very little effect on the morphology of ovary in ELWs. Taken together, our data showed that autophagy was induced and required to sustain increased cell proliferation in the midgut of the honey bee during phenotypic plasticity changes.

## 3. Discussion

**Figure 8:**
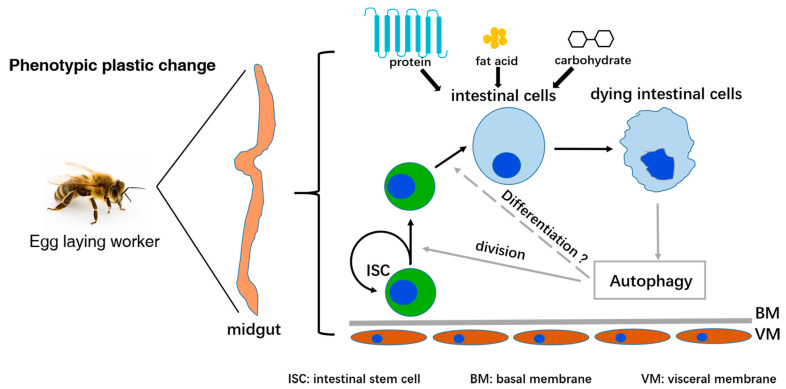
A proposed model of active autophagy in intestines of ELWs during phenotypic plasticity changes. These nutritional absorption-related genes were obviously up-regulated in midguts of ELWs. Autophagy was activated in the midguts of ELWs. Increased cell proliferation was dramatically suppressed by autophagy inhibition. Altogether, autophagy was induced and required to sustain cell proliferation in ELWs’ midguts during phenotypic plasticity changes.

Phenotypic plasticity plays important roles in honeybee; however, it remains unclear whether and how midgut homeostasis in ELWs is affected during plastic changes. Here we show that autophagy is induced and required to sustain increased cell proliferation in the midguts of ELWs during phenotypic plasticity changes (Figure 8).

In the queen-less colony, some of queen-less workers reactivate their ovary and lay haploid eggs [17,18]. In our honey bee breeding center, if the queen was absent for approximate two weeks, about 30 percent of workers in colony would lay haploid eggs, which would develop to drones. Previous studies mainly focused on the hormone from the queen, the nutrition of food, and the cellular signaling [13,79,80]. Here, we compared the genes expression profiles in midguts of ELWs and NWs by RNA-seq, and the analysis results demonstrated that the expressions of some nutrition and autophagy related genes were up-regulated in midguts of ELWs. Autophagy was also increased in the intestines of ELWs. Cellular materials were encapsulated into autophagosomes, which fused with lysosome where these cellular materials were degraded and the nutrients produced were recycled by the cells. With the nutrients derived from increased autophagy, cell proliferation in ELWs’ intestines was significantly activated. Consistently, the body size and mitotic cell number in midguts of larvae fed with RJ were significantly increased compared to those without RJ, further supporting the notion that the nutrition derived from increased autophagy (and/or absorbed from gut epithelium) could promote intestinal cell proliferation. Some intestinal EdU-positive cells with large nucleus may be endopolyploid. Endopolyploidy appears common in midguts and ovary of *Drosophila* and honey bee, and leaves and roots of plants [77,78,81]. Endoreduplication played important functions in organ function [82], the activation of transcription [83], and nutrient storage and cycling [84]. The nutrition level of diet may influence the activity of cell proliferation and tissue homeostasis in the honey bee midgut. Collectively, these results suggested that the ELWs in the queen-less colony had different nutritional levels from those NWs in queen-right colony, which led to the phenotypic plasticity change in the end.

In holometabolous insects, many tissues and organs such as the ovary, midgut and fat body undergo a remodeling process during metamorphosis [30,31,85]. Autophagy is induced in conditions such as nutrient deprivation and starvation [86,87]. In addition, increased stem cell division has been demonstrated to drive intestinal growth in response to nutrition level [88]. To respond to environmental stresses, autophagy is induced during intracellular structural remodeling to encapsulate and digest nonfunctional cell components into lysosomes, to recycle nutrients [89], and to sustain various cellular activities and organism development [33]. Autophagy affects stem cell maintenance and differentiation in the remodeling processes [37]. In *Drosophila*, autophagy is indispensable for midgut programmed cell death during metamorphosis [90]. However, the precise mechanisms in the midguts of ELWs were not clear. The expression of autophagic genes, size and number of the autophagosome/autolysosome were significantly increased in ELWs’ intestines. These results suggested that autophagy-induced functions to provide sufficient nutrient for intestinal homeostasis alterations in ELWs during plastic changes. Supporting this, cell proliferation was almost completely suppressed when ELWs were treated with the autophagy inhibitors. More cells underwent death in the midguts of ELWs. Autophagy may have participated in cell turnover by apoptosis or autophagic cell death. Together, these results revealed that autophagy was essential in sustaining the increased cell proliferation in the midgut of honey bee during phenotypic plasticity changes. It will be interesting to investigate how autophagy is induced during phenotypic plasticity in the future. Meanwhile, further studies on the mechanisms in QLWs midguts will likely provide novel insights into the cross-talk between the ovary and midgut during plastic changes.

## 4. Materials and Methods

### 4.1. Honey Bees

Honey bees (*Apis mellifera*) were collected from the Institute of Apicultural Research (IAR), Chinese Academy of Agricultural Sciences (CAAS), Beijing, P.R. China. To collect NWs, the new emerged bees were marked in six queen-right colonies according to the methods described by Feng [91]. The NWs were collected at 14 days post emergence. The NEQs, reared in artificial queen-cells, were young queens within 12 h post emergence in six queen-right colonies. Newly emerged queen and nurse workers were collected, then dissected following Kristen N. Ward’s methods [49]. To collect ELWs, the queen was artificially removed from the colony, and the new emerged bees were marked according to the methods described by Feng [91]. After 2–4 weeks, a part of the worker bees have developed functional ovary, and laid unfertilized eggs [18]. The ELWs were 21 days post emergence, and with fully developed eggs in their ovaries in six queen-less colonies. These ELWs were then collected as Elizabeth J. Duncan’s description and dissected [18].

### 4.2. RNA Sequencing (RNA-Seq)

Total RNA was extracted from the midguts of NWs, ELWs, and NEQs (pool of five midguts) with TRIzol reagent (Invitrogen, Carlsbad, CA, USA) following the manufacturer’s instructions. The quality of the RNA was measured using an Agilent 2100 Bioanalyzer (Agilent Technologies, Santa Clara, CA, USA).

RNA-sequencing libraries were generated with the MGIEasy RNA Library Preparation Kit from BGI (BGI, Shenzhen, Guangdong, P.R. China) following the manufacturer’s recommendations, and the BGISEQ-500 sequencing platform was used for transcriptome sequencing.

### 4.3. RNA-Seq Data Analysis

The obtained raw sequencing data were processed to remove low-quality sequences and adaptor by SOAPnuke (Version 2.1.7) before downstream analyses. Clean reads were mapped to the reference genome of *Apis mellifera* from NCBI (Version: Amel_HAv3.1; refseq accession: GCF_003254395.2) and sorted by coordinate using STAR (Version 2.7.9a). A program named featureCounts (Version 2.0.0) from the Subread package was used to analyze the profile of gene expression. The edgeR package (Version 3.28.1) was used for gene differential expression analysis. Differential expression calculation was based on TPM (transcripts per kilobase per million mapped reads) and a negative binomial distribution model; the false discovery rate (FDR) control method was used to identify the threshold of the P-value. In this case, the screening criteria of significantly differentially expressed genes (DEG) were FDR < 0.05 and |log_2_FC| ≥ 1.

Genes of *A. mellifera* were annotated in the EggNOG database (Version 5.0) var python script emapper.py with “-d euk --tax_scope 50557” to hit Gene Ontology (GO) terms and Kyoto Encyclopedia of Genes and Genomes (KEGG) pathways used as the background in enriched analysis. DEG dataset enrichment in GO and KEGG was performed by R package clusterProfiler (Version 3.14.3).

### 4.4. Protein-Protein Interaction Network Analysis

All DEGs were firstly to identify their orthologous in *Drosophila melanogaster* using OrthoMCL pipeline (version 2.0.9) with default parameters. Then protein-protein interaction (PPI) relationships among DEGs were obtained by mapping orthologous using the STRING (Version 11.5, https://string-db.org, accessed on 1 November 2022) protein network database plugin to construct a PPI network, and visualized by Cytoscape (Version 3.9.1).

### 4.5. Quantitative Real-Time PCR (qRT-PCR)

The relative expression levels of DEGs and autophagy genes in midguts were analyzed by qRT-PCR. Total RNAs were extracted from midguts of NWs, ELWs, and NEQs (pool of five midguts) using the RNA Easy Fast Tissue/Cell kit (Tiangen, DP451) according to the manufacturer’s instructions. The purity of the RNA was assessed using a NanoDrop 2000 spectrophotometer (Thermo Fisher Scientific, Waltham, MA, USA) at 260/280 nm, and RNA integrity was screened by 1.5% (*w*/*v*) agarose gel electrophoresis. cDNA was synthesized from 2 µg of the purified RNA using the FastKing gDNA Dispelling RT SuperMix (Tiangen, KR118-01) following manufacturer’s instructions. The qRT-PCR analysis was performed on a Roche Lightcyc480 machine (Roche, Hercules) using the SuperReal PreMix Plus (Tiangen, FP205-02) containing Hotmaster Taq DNA polymerase and SYBR solution. The qPCR conditions were as follows: 95 °C for 30 s, followed by 40 cycles of 95 °C for 5 s, and 63 °C for 1 min. The qRT-PCR was performed in duplicate on each of three independent biological replicates. All results are presented as Mean ± SEM of the biological replicates. Generation of specific PCR products was confirmed by melting curve analysis. The ribosomal protein 49 (*rp49*, also known as *rpl32*), was used as the normalization control [92]. The primers of differentially expressed genes for qRT-PCR were designed with Primer-BLAST (https://www.ncbi.nlm.nih.gov/tools/primer-blast/index.cgi, accessed on 1 May 2022). The primer sequences were showed in Supplementary Materials (Table S3).

### 4.6. Rearing of Honey Bee Larvae with RJ and without RJ under Laboratory Conditions

To gain first instar larvae, empty combs were placed for the queen laying eggs inside a colony. After 24 h, the queen was put in a cage for the subsequent 72 h. At the first day (D-1), first instar larvae were collected from three different colonies, grafted into 24-well microtiter plates with V.S. diet [93], and transferred to an incubator (34 °C, 96% RH) [9]. The second day (D-2), larvae were also reared with fresh V.S. diet. The components of V.S. diet are composed of 50% fresh-frozen commercial RJ, 6% glucose, 6% fructose, 1% yeast extract and 37% ddH_2_O [93]. D-3 diet: 55% fresh-frozen commercial RJ, 4% glucose, 4% fructose, 0.5% yeast extract and 36.5% ddH_2_O. D-4, -5, and -6 diet for the honey bee larvae with or without RJ was shown in Supplementary Materials (Table S4).

### 4.7. Immunostaining and Fluorescence Microscopy

Immunostaining was performed as previously described [94]. Briefly, the intestines of NWs, ELWs, and NEQs, and defecating larvae were dissected in 1 × PBS (Solarbia, P1000), and fixed in 4% paraformaldehyde for 25 min at room temperature. Samples were rinsed, washed with 0.1% Triton X-100 in 1 × PBS (1 × PBT) for 2 × 5 min and blocked in 3% BSA in 1 × PBT for 20 min. Primary antibodies were added to the samples and incubated at 4 °C overnight. Primary antibodies for immunohistochemistry were used as follows: rabbit anti-PH3 (anti-phospho-histone H3, Cell Signal Technology, 9701S, 1:200), and rabbit anti-LC3 (Abcam, ab128025, 1:500). The primary antibodies were detected by fluorescent conjugated secondary antibodies from Jackson Immuno Research Laboratories. Secondary antibodies were incubated for 2 h at room temperature, and DAPI (Sigma, D9542, 1 μg/mL) was added after secondary antibody staining. Nuclei were labeled with DAPI. The samples were mounted in mounting medium (70% glycerol containing 2.5% 1, 4-diazabicyclo [2.2.2] octane [DABCO, Sigma, D27802]). Confocal fluorescence imaging was performed with a Leica SP8 laser-scanning microscope (Leica), and all images were processed using Adobe Photoshop (Version CS6) and Adobe Illustrator (Version CS6).

### 4.8. EdU Labeling of Proliferation Cell

EdU Detection—EdU (5-Ethynyl-2’-deoxyuridine, EdU) is a thymidine analog that is incorporated into the DNA of dividing cells to indicate DNA synthesis [95]. The Click-iT EdU cell proliferation Assay Kit (Invitrogen, C10337) was used according to the manufacturer’s protocol and as previously described. Proliferation of enteroid crypts in larvae reared with and without RJ was measured using EdU to label cells in the S phase of the cell cycle. EdU reagent (10 μM) was added into the D-4 diet, and fed honey bee larvae for 24 h. EdU reagent (10 μM) was mixed well with honey to feed NWs and ELWs for 24 h. The midguts of larvae, NWs and ELWs were dissected in 1 × PBS, and fixed in 4% paraformaldehyde for 25 min at room temperature. After rinsing twice with 3% Bovine serum albumin (BSA) in PBS, the samples were incubated in 0.5% Triton X-100 in PBS for 20 min at room temperature, and then washed once with 3% BSA in PBS. These midguts were incubated with freshly arranged Click-iT reaction cocktail containing azide-conjugated Alexa Fluor 488 for 30 min at room temperature, protected from light. The samples were washed once with 3% BSA in PBS, and then incubated with 1 μg/ml DAPI for 30 min at room temperature in the dark. These midguts were mounted in mounting medium.

### 4.9. Transmission Electron Microscopy (TEM)

For transmission electron microscopy analysis, honey bee midguts were dissected and immediately fixed in 2.5% glutaraldehyde in PBS for 24 h. The midguts were washed in PBS, post-fixed in 1% osmium tetroxide, and washed in PBS. Graded alcohol series were thereafter used for dehydration. Additionally, these midguts were embedded in Epon-812 resin (Sigma-Aldrich, St. Louis, MI, USA, 45345). Ultrathin sections (80–100 nm) were cut with an ultramicrotome (Leica M80, Wetzlar, Germany), and stained with uranyl acetate and lead citrate. Images were taken on a transmission electron microscope (Hitachi H-7500, Tokyo, Japan) in the Institute of Food Science and Technology, CAAS. All images were processed using Adobe Photoshop and Adobe Illustrator.

### 4.10. TUNEL Staining

TdT-mediated dUTP-Nick-End-Labelling (TUNEL) in situ cell detection kit (TMR Red, 12156792910, Roche, Germany) was used to detect the apoptosis cell in midguts of NWs and ELWs. Briefly, tissues were dissected and fixed as mentioned above. Samples were then washed 3 times with PBS. Tissues were incubated in permeabilisation solution (0.1% Triton X-100 in 0.1% sodium citrate, freshly prepared) for 2 min on ice and then washed 3 times with PBS. Samples were then incubated with TUNEL reaction mix for 60 min at 37 °C in a humidified chamber. DAPI was added after TUNEL reaction. The samples were mounted in mounting medium (70% glycerol containing 2.5% 1, 4-diazabicyclo [2.2.2] octane). Confocal fluorescence imaging was performed with a Leica SP8 laser-scanning microscope (Leica), and all images were processed using Adobe Photoshop and Adobe Illustrator.

### 4.11. Effects of Autophagy Inhibitors on Cell Proliferation of ELWs’ Midguts

Autophagy inhibitors were employed to explore the function of autophagy in intestinal cell proliferation of ELWs and NWs. The 3-Methyladenine (3-MA) is an autophagy inhibitor which inhibits autophagic signaling by blocking the VPS34/PI3 kinase complex [39]. Bafilomycin A1 (BAF), which is a vacuolar-type H^+^-translocating ATPase inhibitor, can prevent the fusion of autophagosomes with the vacuole [96,97]. In a previous report, whiteflies were fed with 3-MA (1 µM) or BAF (10 nM) to examine the impacts of autophagy on tomato yellow leaf curl virus (TYLCV) infection [98]. According to the weight of whiteflies and ELWs, 3-MA (MedChemExpress, HY-19312, 3 µM) and BAF (MedChemExpress, HY-100558, 30 nM) were used to investigate the effects of autophagy on intestinal cell proliferation. ELWs were fed with 3-MA or BAF in 50-mm diameter cylindrical containers for 48 h. NWs were also fed with 3-MA in 50-mm diameter cylindrical containers for 48 h. The control groups of ELWs and NWs were fed with honey which was autophagy-inhibitor free.

### 4.12. Statistical Analysis

To determine the number of EdU^+^ and PH3^+^ cells in midguts, confocal images of 40 × lens/1.0 zoom of honey bee larvae, NWs, ELWs, and NEQs were acquired. The number of EdU^+^, PH3^+^, TUNEL^+^, and total cells were counted in five random fields from the middle of midguts, and the statistical data were obtained from more than ten midguts. The number and percentage of EdU^+^, PH3^+^ and TUNEL^+^ cells were performed using the Student’s *t*-test, and the data were presented as Mean ± SEM. The graphs were created with GraphPad Prism (Version 8.0.2) and further modified using Adobe Illustrator. * *p* < 0.05; ** *p* < 0.01.

To determine the area/size of the LC3 spots, 40 × lens/2.0 zoom confocal images from the midguts of NWs, ELWs, and NEQs were acquired. These images were captured in five random fields from the middle of midguts, and the statistical data were obtained from more than ten midguts. The area/size of the LC3 spots was measured with Image Pro Plus software (Version 6.0.0.260). Statistical analysis of the LC3 puncta was performed using one way ANOVA/Dunn’s Method, and the data were presented as Mean ± SEM. The graphs were created with GraphPad Prism and further modified using Adobe Illustrator. * *p* < 0.05.

## 5. Conclusions

Our data suggested that autophagy was induced and required to sustain cell proliferation in ELWs’ midguts. The current research revealed the critical role of autophagy playing in the intestines during phenotypic plasticity changes.

## Data Availability

The datasets presented in this study can be found in online repositories. The names of the repository and accession number can be found here: https://www.ncbi.nlm.nih.gov/sra/PRJNA904516, accessed on 31 March 2023, PRJNA904516.

## Supplementary Materials

The following supporting information can be downloaded at: https://www.mdpi.com/article/10.3390/ijms24031926/s1.
