# Supplementary material for: Autophagy Is Required to Sustain Increased Intestinal Cell Proliferation during Phenotypic Plasticity Changes in Honey Bee (Apis mellifera)
Source: Int J Mol Sci. 2023 Jan 18;24(3):1926. doi: 10.3390/ijms24031926 (PMC9916008; doi:10.3390/ijms24031926)
Supplement: Supplementary file 1 [file ijms-24-01926-s001.zip › ijms-2095817-supplementary.pdf]

Supplementary materials

Autophagy is Required to Sustain Increased Intestinal Cell Proliferation During Phenotypic Plasticity Changes in Honeybee (*Apis mellifera*)

Yueqin Guo [1,†], Ruoyang Hu [1,†], Naikang Li [1], Nannan Li [1], Jiangli Wu [1], Huimin Yu [1], Jing Tan [1], Zhouhua Li [2,*] and Shufa Xu [1,*]

Table S1. List of genes which were differentially expressed between nurse workers (NWs) and egg laying workers (ELWs) midguts.

genes	logFC	logCPM	PValue	FDR	Sig.	NW_r1_Count	NW_r2_Count	NW_r3_Count	ELW_r1_Count	ELW_r2_Count	ELW_r3_Count	NW_r1_TPM	NW_r2_TPM	NW_r3_TPM	ELW_r1_TPM	ELW_r2_TPM	ELW_r3_TPM	NW_r1_FPKM	NW_r2_FPKM	NW_r3_FPKM	ELW_r1_FPKM	ELW_r2_FPKM	ELW_r3_FPKM
gene-Pla2	11.01645017	5.824182696	3.01E-13	1.31E-09	Up	2	0	0	4486	226	73	0.359109097	0	0	557.8143555	120.8633408	25.70138909	0.265457503	0	0	447.116555	62.43278455	16.12710134
gene-LOC724436	6.843283817	2.833339058	5.75E-09	7.15E-06	Up	2	1	2	501	38	8	0.30774399	0.137188788	0.173244459	53.38648364	17.4153788	2.413720041	0.227487835	0.102635523	0.142041057	42.79197983	8.996032916	1.514560461
gene-LOC411012	2.661589031	5.660916248	8.71E-05	0.00680518	Up	173	210	244	498	626	307	12.11911864	13.11605602	9.622424989	24.15951897	130.6137839	42.16971194	8.958589461	9.812560432	7.88931098	19.36508228	67.46944255	26.46064054
gene-LOC408577	3.010935826	9.393168453	0.000144442	0.008346066	Up	2736	2473	1618	3767	8195	5649	29.15158019	23.49249584	9.704972282	27.7955732	260.0666779	118.0198091	21.54917753	17.57552231	7.956990518	22.27956454	134.3392195	74.05504097
gene-LOC550818	3.13710296	9.258308675	0.000250025	0.011180276	Up	2425	1927	1377	2386	7463	5648	54.83535523	38.8498787	17.52881803	37.36400005	502.6342796	250.4268735	40.53491431	29.06489436	14.37166793	29.94914494	259.6391716	157.1377933
gene-LOC552216	1.616640315	3.14998835	0.000261916	0.011337979	Up	63	54	78	195	56	71	2.356795974	1.801085095	1.642652939	5.05184545	6.239635342	5.208073588	1.742170219	1.347452033	1.346791467	4.049310872	3.223126271	3.267960741
gene-	2.415388799	4.826780115	0.00033	0.0129	Up	139	149	124	201	326	220	1.960788901	1.873964426	0.984708074	1.963568713	13.6969	6.08521	1.449437	1.401975499	0.807350354	1.573900115	7.075245188	3.818350262

LOC408271			7153	2319												3466	666	315					
gene-	2.492639973	9.050664876	0.00036	0.0135	Up	2988	2830	1577	3759	4783	6134	104.7477174	88.45236399	31.12185265	91.25786555	499.406	421.643	77.43069	66.17417355	25.51643417	73.14781712	257.971786	264.5725542
LOC550930			7134	53018												3955	1716	65					
gene-	2.447796789	7.626348352	0.00051	0.0161	Up	1030	794	941	1233	1864	2189	35.3465954	24.29348948	18.17899045	29.30268705	190.522	147.296	26.12860	18.17477246	14.9047365	23.48759289	98.41564133	92.42578958
LOC409286			3002	72577												388	847	279					
gene-	2.374225088	8.941574128	0.00176	0.0304	Up	3004	2519	1771	2646	5453	4182	64.38567291	48.13672198	21.36867756	39.27472536	348.108	175.756	47.59461	36.01269261	17.51992276	31.48068839	179.8177931	110.28346
LOC410795			9805	32949												4396	2041	707					
gene-	2.158522707	7.427061155	0.00188	0.0318	Up	1303	1188	445	1353	1618	1667	37.50538584	30.48770242	7.21072623	26.97000487	138.713	94.0853	27.72440	22.80887045	5.911988058	21.61782956	71.65320058	59.03667172
LOC409791			9457	60779												1018	8077	508					
gene-	2.339836464	6.762071425	0.00208	0.0340	Up	586	552	497	544	1301	779	13.91742507	11.68851632	6.644884529	8.94734496	92.0297	36.2773	10.28791	8.744570214	5.448061226	7.171751705	47.53860431	22.76332909
LOC411630			3646	14635												657	9205	79					
gene-	2.351870247	9.053197198	0.00217	0.0350	Up	3518	2637	1822	2685	5570	5041	84.23120363	56.29195548	24.55813137	44.52003201	397.211	236.663	62.26465	42.1138957	20.13491773	35.68506824	205.1824549	148.5015247
LOC726289			7344	83463												7719	4515	766					
gene-	-1.608471437	4.542650604	0.00235	0.0364	Dow	338	401	799	199	24	85	32.95116025	34.8543845	43.8500976	13.43510589	6.96875	16.2483	24.35787	26.07573144	35.95216974	10.76892017	3.599756359	10.19553408
LOC411376			6253	7289	n											1799	8728	005					
gene-	-2.141788895	3.392501398	0.00303	0.0418	Dow	124	158	447	120	8	9	5.480679673	6.226282989	11.12218288	3.673059698	1.05315	0.77999	4.051380	4.658090666	9.11894451	2.944144019	0.544014627	0.489431918
LOC413263			4211	39409	n											5417	6348	354					
gene-	-2.35047099	7.679669314	0.00172	0.0299	Dow	2849	3569	8644	2247	85	253	195.1597874	217.9733853	333.3361533	106.5945415	17.3422	33.9825	144.2643	163.073184	273.2983191	85.44094232	8.958280723	21.32335081
LOC726315			1744	01983	n											945	3191	204					

Note: The genes were ranked by the log2 Fold-change (LFC). Genes were identified by their BeeBase gene identifier. SEM is the standard error of the mean of the log2 Fold-change. FDR is the False Discovery Rate.

Table S2. KEGG analysis of autophagy-related pathways in midguts of ELWs.

ID	Description
ko04020	Calcium signaling pathway
ko04611	Platelet activation
ko04933	AGE-RAGE signaling pathway in diabetic complications
ko04925	Aldosterone synthesis and secretion
ko04916	Melanogenesis
ko04972	Pancreatic secretion
ko04912	GnRH signaling pathway
ko04071	Sphingolipid signaling pathway
ko04024	cAMP signaling pathway
ko04919	Thyroid hormone signaling pathway
ko04010	MAPK signaling pathway
ko04911	Insulin secretion

Table S3. Primer sequences for qRT-PCR analysis.

Number	Gene	Gene ID	Forward primer	Reverse primer	Product length (bp)
1	*pla2*	406141	TGCTCTCGCGTACTCTGTGA	TCCTATCCCTGATTTGCCAT	206
2	*phospholipase A2-like*	724436	CCTGTTCCTCCACGTTTCTG	ATAAGCTTGCGTTCCACAGA	245
3	*rapgap1*	408271	CAAGACACGGTCAAACAGGC	GAAGTGGCTGGCGATCATAT	206
4	*transcription factor ap-1*	726289	TTTTCTCCCGGAGTGTCG	AATCCCGATTTTCCCGAA	198
5	*sphingomyelin phosphodiesterase 1*	726315	AGAACCAGTTTGTTGCCGA	CTTGATGTGTATCCTTCATGTGA	146
6	*putative inorganic phosphate cotransporter*	413263	GGTGAATGGGTACCAGCGA	CATGAACGATAACACCGACG	241
7	*estrogen sulfotransferase*	411376	AATGACTCTTGAACCACCGA	GACCTACAAGCACCCAACCT	104
8	*atg1*	552571	TTCAGAACGTGCCTCACAGA	AGTGCTGCTGATTGACACATT	122
9	*atg2*	726497	TACGGTACCAAGTTCTGGCG	AGTTACTAATGTATTGCCGTCTTTT	145
10	*atg3*	552315	GGCTTCAGTTCATCCATGCAG	ACTCCAAGTTCACGACCACC	84
11	*atg4b*	552197	TCCTTCTGTAGCACTTTGTTTCT	GCAACATCTTCAACCGACCA	153
12	*atg5*	551057	CCACTCTGCACAGATAAGATACGA	TCTGGAAACCTATCAAAGTGGACA	197
13	*atg6*	408834	GCTGAACTTTCACTTCCCATC	TGTTTCCCCAGAGTCACCAAC	150
14	*atg8*	411870	AGGGCAGCACAAACCAATGT	GCAACCCTTTGTGCAAATGAA	216
15	*atg10*	726781	CCAATGCTTTGCCAGACAAGT	TCCATTATTCTCAGATCACTCCTGT	113

16	*atg12*	113219359	TGTTATACACCTACTCAGGAGAATC	TGTACGTTAAGTTTCAAATGCCCG	95
17	*atg13*	551384	CCTCCTCCAGTCTCAACAGC	CGGCGAAAGGAGTTTTCAAAT	211
18	*rp49*	406099	CGTCATATGTTGCCAACTGGT	TTGAGCACGTTCAACAATGG	150

Table S4. D-4, -5, and -6 diet without or with RJ for honeybee larvae.

	Day	RJ (g)	glucose (g)	fructose (g)	yeast extract (g)	ddH_2O (mL)
	D-4	0	8	4	0.5	47.5
**Without RJ**	D-5	0	8	4	0.5	47.5
	D-6	0	8	4	0.5	47.5
	D-4	40	8	4	0.5	47.5
**With RJ**	D-5	40	8	4	0.5	47.5
	D-6	40	8	4	0.5	47.5

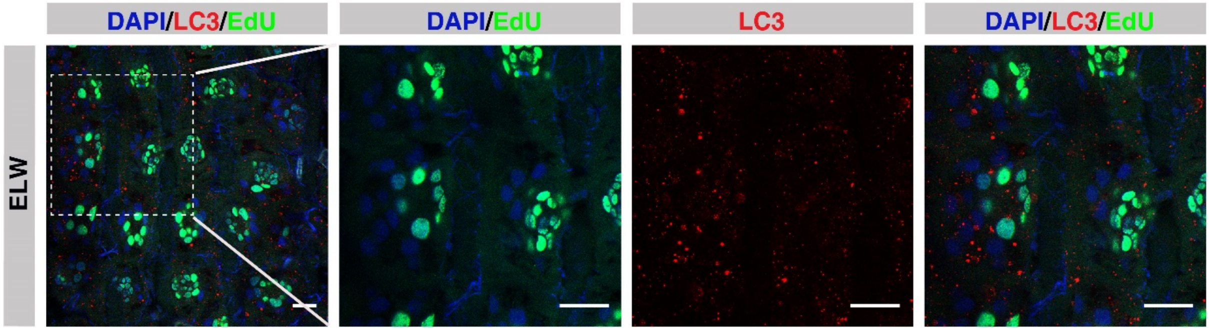

Figure S1. The colocalization of EdU and LC3 in midguts of ELWs. In all the panels except graphs, blue indicates DAPI staining, green indicates EdU positive cells, and red indicates LC3 staining. In replicating chromosomes, EdU is integrated into newly synthesized DNA. The signal of LC3 is almost dispersed in cytoplasm of ELWs midguts. Scale bars, 25 μm.

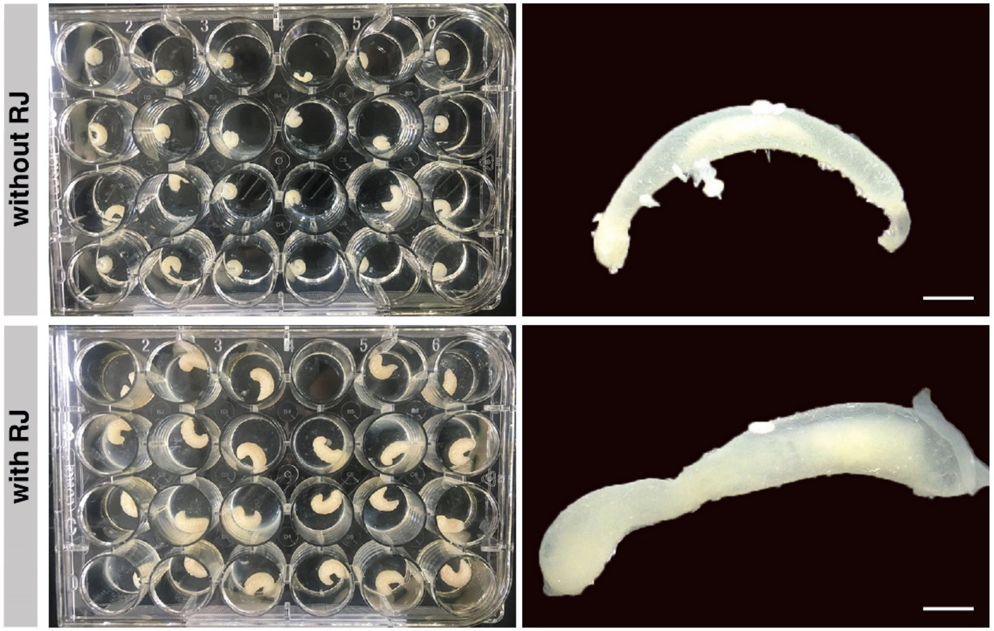

Figure S2. RJ affects the larvae body and midgut sizes. The body and midgut sizes of larvae reared with RJ were much larger than those without RJ. Scale bars, 1 mm.

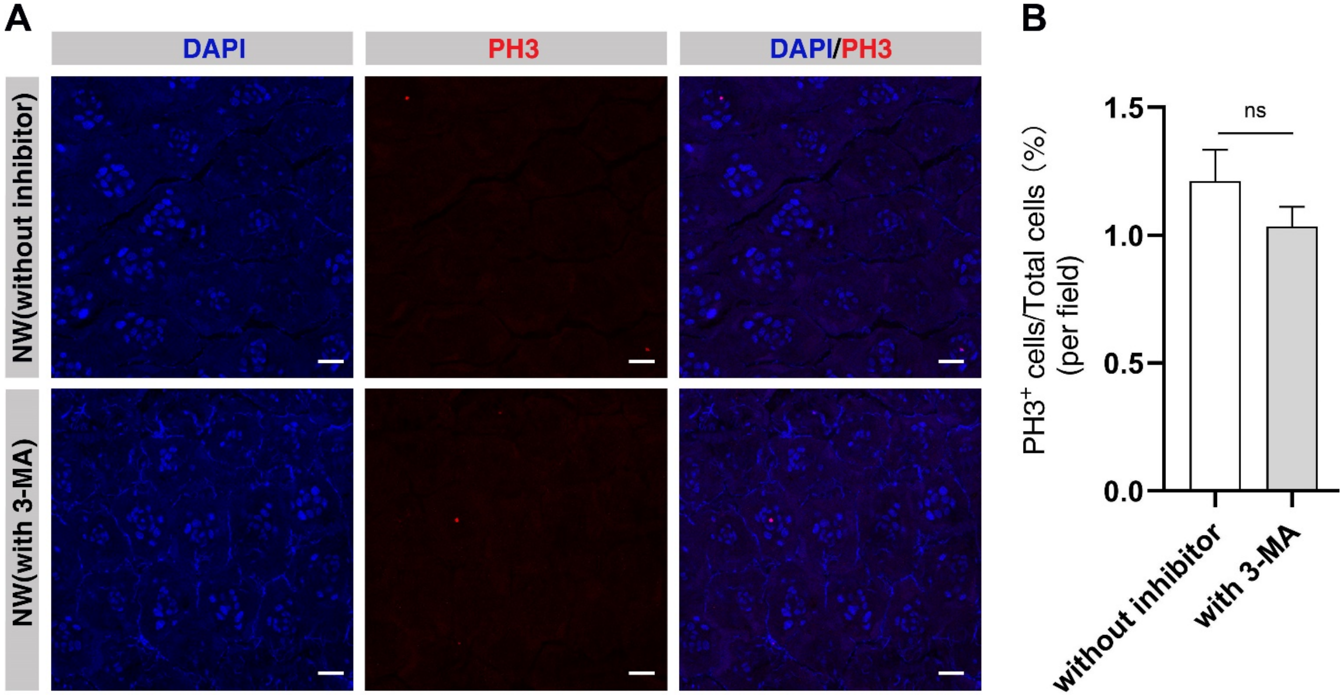

Figure S3. No obvious difference in intestinal cell proliferation of NWs treated with or without 3–MA. (A) Detection of cell proliferation with PH3 antibody in the midguts of NWs which were fed with or without 3–MA. In all the panels except graphs, blue indicates DAPI staining, and red indicates PH3 staining. Scale bars, 25 μm. (B) The percentage of PH3 positive cells in the midguts of NWs fed with or without 3-MA (with 3–MA, 1.03 ± 0.09%, n = 10 intestines; without inhibitor, 1.21 ± 0.13%, n = 10 intestines). Means ± SEM are shown. Significant differences between the midguts of NWs fed with or without 3–MA were determined by one way ANOVA/Dunn's Method. There is no significant differences between the midguts of NWs treated with or without 3-MA. ns = not significant.
